# Patient-Derived Xenograft Models of Non-Small Cell Lung Cancer and Their Potential Utility in Personalized Medicine

**DOI:** 10.3389/fonc.2017.00002

**Published:** 2017-01-19

**Authors:** Katherine M. Morgan, Gregory M. Riedlinger, Jeffrey Rosenfeld, Shridar Ganesan, Sharon R. Pine

**Affiliations:** ^1^Rutgers Cancer Institute of New Jersey, Rutgers, The State University of New Jersey, New Brunswick, NJ, USA; ^2^Department of Pharmacology, Robert Wood Johnson Medical School, Rutgers, The State University of New Jersey, New Brunswick, NJ, USA; ^3^Department of Pathology and Laboratory Medicine, Robert Wood Johnson Medical School, Rutgers, The State University of New Jersey, New Brunswick, NJ, USA; ^4^Department of Medicine, Robert Wood Johnson Medical School, Rutgers, The State University of New Jersey, New Brunswick, NJ, USA

**Keywords:** patient-derived xenograft, lung cancer, personalized medicine, precision medicine, preclinical trial

## Abstract

Traditional preclinical studies of cancer therapeutics have relied on the use of established human cell lines that have been adapted to grow in the laboratory and, therefore, may deviate from the cancer they were meant to represent. With the emphasis of cancer drug development shifting from non-specific cytotoxic agents to rationally designed molecularly targeted therapies or immunotherapy comes the need for better models with predictive value regarding therapeutic activity and response in clinical trials. Recently, the diversity and accessibility of immunodeficient mouse strains has greatly enhanced the production and utility of patient-derived xenograft (PDX) models for many tumor types, including non-small cell lung cancer (NSCLC). Combined with next-generation sequencing, NSCLC PDX mouse models offer an exciting tool for drug development and for studying targeted therapies while utilizing patient samples with the hope of eventually aiding in clinical decision-making. Here, we describe NSCLC PDX mouse models generated by us and others, their ability to reflect the parental tumors’ histomorphological characteristics, as well as the effect of clonal selection and evolution on maintaining genomic integrity in low-passage PDXs compared to the donor tissue. We also raise vital questions regarding the practical utility of PDX and humanized PDX models in predicting patient response to therapy and make recommendations for addressing those questions. Once collaborations and standardized xenotransplantation and data management methods are established, NSCLC PDX mouse models have the potential to be universal and invaluable as a preclinical tool that guides clinical trials and standard therapeutic decisions.

## Introduction

In 1969, Rygaard and Povlsen reported successful serial passages of a malignant sigmoid colon carcinoma implanted subcutaneously into nude mice. Histological integrity was maintained during the transfers with regard to degree of differentiation, stromal components, and mucoid production compared to that of the primary tumor (1). This was the first reported human solid tumor PDX in mice. Since then, the variety and availability of immunodeficient host strains has greatly increased, leading to improved tumor engraftment rates and the subsequent widespread use of PDX mouse models in academia and industry.

**Figure 1:**
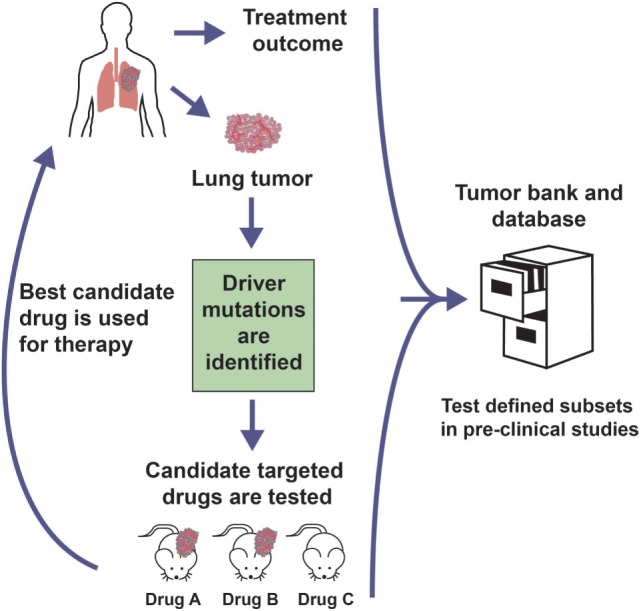
**Personalized medicine clinical trial approach with PDX models**. Genomic analysis of a patient tumor identifies potential therapeutically targetable mutations. Rationally chosen molecularly targeted agents against the identified driver mutation are tested in PDX models generated from the patient sample. The most promising agent could be administered to the patient, usually at the time of tumor recurrence or initial treatment failure. The treatment outcome and preclinical trial data are banked in order to inform future studies.

PDX mouse models can potentially be used to (i) gain a better understanding of cancer biology, (ii) investigate novel anticancer treatments while considering both a patient-derived tumor tissue and an *in vivo* setting, (iii) study biomarkers of therapy response and resistance, and (iv) develop personalized therapeutic regimens (2–4) [reviewed in Ref. (5, 6)]. The recent popularity of PDX models has paralleled advances in high-throughput next-generation sequencing, which has provided a window into the complexities of human cancer and demonstrated the need to distinguish driver alterations from passenger events within the heavy mutational load. Subsequent characterization of the landscape of genetic mutations and copy number variations has facilitated the identification of “druggable” driver mutations. A number of novel drugs that target oncogenic drivers have been developed and entered the clinic. However, this development was limited in part by the use of human cancer cell lines that had been propagated in the laboratory for decades, resulting in major and irreversible biological changes, including gain or loss of genetic information, metastatic ability, and stem cell populations, which leads to divergence from the tumor they were meant to represent (5, 7–9), compromising their predictive value with regards to therapeutic activity. The recent collection and utilization of large PDX tumor banks has offered an exciting tool for potentially maximizing the efforts and ultimate success of drug development given their ability to represent and maintain biological integrity and tumor heterogeneity present in the clinic due to their lack of *in vitro* culture and low-passage xenoengraftment. Clinically and molecularly annotated PDXs that fit within defined genetic subsets could potentially be procured from various sources and used to predict the response of patient tumors with similar genetic backgrounds, providing an effective resource to aid in identification of chemoresponsive biomarkers and subsequently target patient populations. As a clinically representative tool that best recapitulates the biological properties of their respective tumor type, PDX mouse models could serve as an important aid in personalized medicine studies as well. Specifically, PDXs could be used as part of co-clinical trials, whereby the model and patient are treated simultaneously (5, 6). Similarly, precision medicine-directed clinical trials could utilize PDX models, whereby the xenografted animals are treated with rationally chosen targeted therapies and the most effective option is given to the patient, usually in the setting of tumor recurrence or after the initial therapy proved ineffective (Figure 1). These approaches of tailoring cancer therapy based on PDX models could lead to better informed treatment decisions, which potentially increases the success rate of clinical trials and ultimately patient care.

## Patient-Derived Xenograft Models of Non-Small Cell Lung Cancer (NSCLC)

Despite substantial improvements in early detection procedures and targeted therapies, lung cancer is still the leading cause of cancer-related deaths worldwide (10). Front-line treatment of patients with advanced NSCLC historically consisted of radiation and/or standard systemic chemotherapeutic drugs, such as carboplatin and paclitaxel. Recently, the discovery of “actionable” genetic alterations has resulted in the development of targeted therapeutic agents. In particular, the identification of mutations in the gene encoding epidermal growth factor receptor (EGFR) in NSCLC patients with adenocarcinoma has led to the utilization of small-molecule tyrosine kinase inhibitors, such as gefitinib or afatinib specifically for that subtype of patients (11). Moreover, patients with translocations involving the anaplastic lymphoma kinase (ALK) can be treated with ALK inhibitors, such as crizotinib or ceritinib (12, 13). Additionally, crizotinib has also been shown to be effective in advanced NSCLC patients with ROS1 translocations (14). Consequently, according to the College of American Pathologists, the International Association for the Study of Lung Cancer, and the Association of Molecular Pathologists, it is recommended that, whenever feasible, all advanced NSCLC patients with elements of the adenocarcinoma histological subtype should be screened for EGFR mutations and ALK fusions in order to guide therapy selection (15). Immune checkpoint inhibitors that target either PD-1 or PD-L1 have also proven effective. Based on the data from a seminal trial (16), a monoclonal antibody targeting PD-1, pembrolizumab, has recently been approved by the US Food and Drug Administration for use as a front-line therapy for advanced NSCLC patients without activating EGFR mutations or ALK fusions, but expressing PD-L1. Additional therapeutic agents, including compounds that could target oncogenic alterations in *KRAS, PIK3CA, AKT1*, or *HER2*, as well as MET amplification and RET fusions, are being actively developed and tested. It is expected that PDX models from primary or metastatic NSCLC tumors will facilitate the preclinical testing of these new compounds, which may hasten their potential usage in the clinic.

Primary or metastatic NSCLC tumors have been utilized to establish orthotopic or heterotopic (subcutaneous, subrenal capsule) PDX murine models from multiple labs, with reported engraftment rates ranging between 23 and 90% (2, 3, 17–20). Moreover, NSCLC PDXs have been generated by consortium members of the EurOPDX,^1^ PRoXe (21),^2^ the NCI,^3^ and the Jackson Laboratory,^4^ as well as by for-profit companies, including Champions Oncology, Novartis (22), and OncoMed Pharmaceuticals, among others. In general, squamous cell carcinoma has demonstrated a higher engraftment rate than adenocarcinoma (17, 18). Specifically, Russo’s group determined that PDXs derived from adenocarcinoma had decreased Ki67 staining and lower expression of stem-cell-related genes (*SOX2* and *ALDH1A1*), which could contribute to the reduced engraftment success (17). The utility of PDX models for human NSCLC depends on the precise reflection of the parental tumors’ pathologic and molecular characteristics. Overall, early-passage PDXs retained many of the mutations or allele frequencies that were present in the original tumor (2, 18, 20, 22). Most of the studies documented that the PDXs had preserved morphological (2, 3, 19, 20) and immunohistochemical (2, 3, 20) features compared to the donor tissue; however, Russo’s group reported that only 60% of the xenografts retained the original tumor morphology at all harvesting points. Specifically, the loss of preserved morphology was observed mainly in adenocarcinoma PDXs that lost their tumor epithelial component within 3 months after engraftment (17). As has been previously reported for numerous other tumor types, established NSCLC PDXs have lost the human stroma and immune cells after repeated passages as well (2, 5, 9, 23, 24). Depletion of human-derived tumor-associated cells within NSCLC PDXs was corroborated in gene expression studies that revealed a downregulation of genes corresponding to cell adhesion and immune response pathways (2).

We have generated a PDX tumor bank from NSCLC patient samples collected through the Rutgers Cancer Institute of New Jersey Biospecimen Repository Service and approved by the Institutional Review Board (IRB) of Rutgers University. We successfully produced 20 PDX models from 66 lung tumors (30% success rate) by means of passaging and expansion through subcutaneous engraftment in NOD *scid* gamma (NOD.*Cg-Prkdc^scid^Il2rg^tm1Wjl^*/SzJ) mice. The animal protocol was approved by the Institutional Animal Care and Use Committee of Rutgers University. In order to characterize and validate our PDX models, we selected nine matched pairs of primary tumors and their corresponding PDXs (at passage three or four) (four adenocarcinoma and five squamous cell carcinoma) to undergo histological evaluation and next-generation sequencing.

Microscopic examination of hematoxylin and eosin (H&E)-stained formalin-fixed paraffin-embedded tissues by a board certified pathologist revealed that the tumor cells from the majority of the early-passage PDXs maintained the morphological features of NSCLC, except for two PDX tumors that transformed into a small cell carcinoma histology (Table 1). Overall, there was a trend toward higher histologic grade in the PDXs compared to the parental tumor. Specifically, out of the six lung tumors that were moderately to well differentiated, four became poorly differentiated or transformed to small cell histology after minimal murine passages (Table 1). This may be attributed to clonal selection and the loss of tumor heterogeneity, as well as the reduction of human stromal components upon passaging through mice. In every case, there was a substantial decrease or loss of human-derived tumor stroma and accessory cells, including normal lung parenchyma, vasculature, immune cells, and fibroblasts.

**Table 1:** **Histological and genetic features of lung tumors and PDXs**.

**ID**	**Subtype**	**Patient tumor**				**PDX**			

		**Differentiation**	**Gene**	**Mutation**	**Frequency**	**Differentiation**	**Gene**	**Mutation**	**Frequency**
	
T-009	SCC^c^	Moderate	SMO	S354L	0.11	Small cell^b^	None	None	
			FGFR1	D161N	0.10				
			SMAD4	R531Q	0.07				
T-020	ADC^d^	Moderate	SMO	V411A	0.06	Moderate	None	None	
			AKT1	R25H	0.08				
			CDH1	T406A	0.06				
T-025	ADC^d^	Moderate–well	KIT	V530I	0.13	Small cell^b^	None	None	
T-035	SCC^c^	Poor	None	None		Poor	ATM	R1768L	0.07
							ATM	S1947F	0.06
T-042	ADC^d^	Moderate	TP53	R273L	0.16	Poor	TP53	R273L	0.99
T-050	SCC^c^	Poor	PIK3CA	Q452L	0.13	Poor	PIK3CA	Q452L	0.15
							TP53	V274L	0.08
T-054	ADC^d^	Moderate	KRAS	G12V	0.11	Poor	KRAS	G12V	0.30
T-065	SCC^c^	Moderate^a^	FLT3	A680V	0.67	Moderate	FLT3	A680V	0.95
			GNA11	V359A	0.06				
T-067	SCC^c^	Poor	MET	L1243I	0.16	Poor	MET	L1243I	0.20
			TP53	V274L	0.56		TP53	V274L	0.99
							NOTCH1	A1701T	0.06

*^a^The evaluation of tumor grade was from a PDX that was passaged once in a mouse*.

*^b^The tumor phenotype transformed into a histology consistent with small cell carcinoma*.

*^c^SCC indicates squamous cell carcinoma*.

*^d^ADC indicates adenocarcinoma*.

Utilizing our panel of matched pairs of primary tumors and their corresponding PDXs, we PCR-enriched the genomic DNA for known mutational hotspots in 50 cancer genes using the ThunderBolts Cancer Panel v7.1 (RainDance Technologies) and then sequenced the targeted DNA on a MiSeq System (Illumina, Inc.) to a coverage of at least 500×. Alignment and variant calling were performed using BaseSpace with default parameters. The variants were filtered using VarSeq (Golden Helix) to exclude all synonymous or benign mutations and to exclude variants with less than 5% minor allele frequency. Only two (T-042 and T-054) of the nine matched pairs had the same mutations detected in both the patient tumor tissue and the low-passage PDX (Table 1). Furthermore, out of the 14 total mutations detected in the primary tumors, only 6 (43%) were detected in the corresponding PDXs. Four additional mutations arose in the early passage PDXs that were not detected in the parental tumor. We examined the raw sequence reads and confirmed the absence of the mutations in the corresponding primary tumors. These data suggest that clonal selection and evolution occur early on when human tumors are propagated in mice. Additionally, the majority of mutations that were detected in both the patient tumors and PDXs had higher mutant allele frequencies in the PDX compared to the donor tissue. These differences may be attributed to loss of stroma and thus an enrichment of tumor cells in the PDX as compared to the primary sample or, in the case of *TP53* in T-042 and T-067, a loss of heterozygosity. An assessment of the percentage of tumor cells in each sample by the pathologist indicated that the increased allele frequencies in the PDX tumors could at least partially be attributed to the increase in tumor cells. The estimated percentage of tumor cells within the primary tumors ranged from 20 to 80% (mean, 59%), whereas the estimated percentages of tumor cells within the PDXs were all “>90%,” with the exception of T-054, which had 60% tumor cells. However, the observed increase in allele frequencies in the PDXs might also be attributed to clonal selection. This evidence for clonal evolution occurring early during passaging in mice needs to be addressed systematically, and the consequence of such evolution on response to targeted therapy needs to be fully defined.

## Patient-Derived Xenografts as Preclinical Models for Personalized Medicine

While many of the NSCLC PDX model studies have utilized subcutaneous implantation for xenoengraftment (2, 18, 25), others have focused on orthotopic sites (19, 26) or the well-vascularized subrenal capsule (3, 17). When compared to the subcutaneous model, orthotopic implantation may maintain improved tumor integrity and demonstrate more phenotypic characteristics, such as metastasis development, but the models have not been directly compared to address if one is a better predictor of response to therapy (5, 27, 28). Moreover, while orthotopic transplantation may more accurately mimic the parental tumors by virtue of replicating aspects of the native microenvironment, this method is technically challenging, labor intensive, and expensive (5), which could impede its universal implementation and, thus, its ultimate utility in preclinical modeling. Recently, an area of active investigation has been the humanized mouse xenograft model, where immune cells, such as hematopoietic stem cells from cord blood or matched from the donor, are co-transplanted into immunodeficient mice along with patient tumor tissue (29). Such models will be needed to test the efficacy of immunotherapy or to study antitumor immunity as well as the involvement of the immune system in responsiveness to chemotherapy.

Overall, the ability of primary samples to engraft is still suboptimal and a PDX cannot be created for every patient (2, 5, 18, 19, 27). Additionally, the high cost and the amount of time needed for implantation, expansion, and drug testing renders prospective co-clinical trials using patient-centric mouse avatars less suitable for use in real-time therapeutic decision-making, especially for patients with advanced or aggressive tumors (5, 6, 9, 25). However, PDX models have the potential to be immensely valuable in preclinical trials whereby the data will be used to guide therapeutic decisions for future patients. Consequently, in the near future, PDX models might serve best as excellent tools for co-clinical trials only in certain circumstances.

On the other hand, PDX models can be used retrospectively to identify therapeutic recommendations for patients who have molecular characteristics similar to those of the donor patient from whom the xenograft was derived (5, 27). Collaborations between groups and networks have been, and should continue to be, developed in which existing PDX model material and data are gathered and shared (Figure 1). For such interactions to be productive, the cataloged information should be derived from standardized xenotransplantation methods, sample validation and data collection procedures, nomenclature, banking of genetic and histomorphological characterization, therapeutic response or resistance reporting, and so on, such that the resource provides all necessary information to obtain the best possible match for the current patient being treated in the clinic. This is imperative given the fact that even morphologically similar cancers are exceedingly heterogeneous at the genomic level, while subject to a unique interplay with stromal components and additional cells found in the tumor microenvironment, which may influence the response to treatment. The EurOPDX Consortium (5) as well as the NCI Patient-Derived Models Repository can serve as prototypes for this initiative as they demonstrate how to successfully bring together multicenter translational and clinical researchers to standardize sample processing and data collection to create a network of annotated PDX models with the primary goal of collaborating on preclinical and co-clinical trials.

To maximize the utility of a PDX model database to inform treatment options, it is well understood that contributors would ideally distribute requested models to investigators for drug testing, while reporting all regimens previously evaluated. However, to be translational, the preclinical standards of success would need to be aligned with the criteria used in the clinic. For example, a statistically significant tumor growth reduction of 70% compared to untreated mice in a preclinical experiment would be seen as 30% tumor growth during treatment in the clinical setting, which is considered progressive disease and treatment failure. Success in patient care is represented by stable disease or regression (24, 27). In addition, we need to take into account the differences in drug metabolism and pharmacokinetics between a mouse versus a human (30). Futhermore, we should keep in mind that the majority of preclinical studies utilize weight-adjusted therapy dosages, while the assessment of drugs at clinically achievable exposures would be better at predicting clinical efficacy (27). In general, it must be remembered that PDXs are models. They are only representations of the real life situation. This notion is often forgotten when the findings are extrapolated to more general conclusions (27) or, as it pertains here, when data from preclinical studies utilizing PDX models is translated in the clinic, often resulting in expensive failures and hasty dismissal of potentially informative data.

An outstanding question in the field is whether or not PDXs must maintain the histopathological and molecular characteristics of the parental tumor to be reliable preclinical models. Our data presented here and the work of others have shown that generation of a PDX model in a murine host leads to clonal selection (18, 23, 27), which may eventually result in a xenograft that differs from the original patient’s tumor. However, the selection of “stronger” clonal subpopulations may represent genetic changes that would eventually occur in the primary lesion, particularly after chemo- and/or radiotherapy, and contribute to tumor survival, metastasis, and targeted treatment efficacy (5, 6, 9, 23), making the utility of these PDX models still viable. One way to test this is to directly compare the genomic landscape between low-passage PDXs and tumor biopsies obtained from a large cohort of patients who have recurrent disease after standard therapy.

In addition to clonal selection, our data and studies reported by others have demonstrated that even in early murine passages, PDX human stromal elements (including cancer-associated fibroblasts, endothelial cells, as well as immune and inflammatory cells) are lost (2, 5, 9, 23). In general, all PDXs will eventually lose the human stromal elements. Thus, the consensus is that a low-passage number is ideal to conserve histological and genetic integrity of the primary tumor (6, 9). However, given that the speed and extent with which this transition from human stroma to murine stroma occurs is still controversial and may be tumor-specific, it is unknown which low passage is the magic number. To combat this loss of stromal components through xenoengraftment, some researchers have taken to using supplemental support matrix, such as Matrigel, or coimplantation of patient-matched fibroblasts, which may improve survival of neoplastic cells and, thus, engraftment rate of implanted tumor samples (23, 27). Similarly, patient-matched humanized PDX models with coengrafted stromal and immune components along with the donor tumor tissue (23, 29), while expensive and labor intensive for general use, may allow researchers and clinicians to both predict and explain tumor response to therapy in instances where the tumor–stroma and tumor–immune interactions must be taken into account.

There is a general consensus that the technical and logistical challenges need to be fully addressed before PDX model systems can be universally utilized to inform clinical decision-making. Any changes in the tumor microenvironment and clonal selection that occurs through xenotransplantation must be considered, measured, and factored into the experimental findings. While PDX models do not perfectly mimic all aspects of human cancer, they are a valuable tool for evaluation of targeted therapies and elucidation of biomarkers for predictive treatment response.

## Ethics Statement

The study was carried out in accordance with the recommendations of the IRB of the Rutgers Cancer Institute of New Jersey. The study was deemed by the Rutgers University IRB to not involve human subjects because (1) the tissues were anonymized through an honest broker’s system with no possible link back to the patient and (2) the tissues were pathological discards. This study was carried out in strict accordance with the Guide for the Care and Use of Laboratory Animals of the National Institutes of Health. The animal protocol was approved by the Institutional Animal Care and Use Committee of Rutgers University.

## Author Contributions

KM designed the study, performed the research, analyzed the data, and wrote the manuscript. GR performed the histological evaluations. JR analyzed the sequencing data. SG analyzed the data and provided advice throughout the study. SP designed the study, performed the research, analyzed the data, revised the manuscript, and supervised the study. All authors discussed the results and commented on the manuscript.

## Conflict of Interest Statement

The authors declare that the research was conducted in the absence of any commercial or financial relationships that could be construed as a potential conflict of interest.

## Footnotes

^1^http://europdx.eu/pdx-collection.html

^2^https://proxesite.wordpress.com/

^3^https://dtp.cancer.gov/organization/btb/tumor_repositories.htm

^4^https://www.jax.org
